# Centering Public Perceptions on Translating AI Into Clinical Practice: Patient and Public Involvement and Engagement Consultation Focus Group Study

**DOI:** 10.2196/49303

**Published:** 2023-09-26

**Authors:** William Lammons, Milou Silkens, Jamie Hunter, Sudhir Shah, Charitini Stavropoulou

**Affiliations:** 1 National Institute of Health and Care Research, Applied Research Collaboration North Thames Department of Applied Health Research University College London London United Kingdom; 2 Erasmus School of Health Policy and Management Erasmus University Rotterdam Netherlands; 3 Centre for Healthcare Innovation Research City University of London London United Kingdom; 4 Public co-author National Institute of Health and Care Research, Applied Research Collaboration North West Coast, Department of Health Services Research The University of Liverpool Liverpool United Kingdom; 5 Public co-author National Institute of Health and Care Research, Applied Research Collaboration North Thames, Department of Applied Health Research University College London London United Kingdom

**Keywords:** acceptance, AI in health care, AI, artificial intelligence, health care research, health care, patient and public engagement and involvement, patient engagement, public engagement, transition

## Abstract

**Background:**

Artificial intelligence (AI) is widely considered to be the new technical advancement capable of a large-scale modernization of health care. Considering AI’s potential impact on the clinician-patient relationship, health care provision, and health care systems more widely, patients and the wider public should be a part of the development, implementation, and embedding of AI applications in health care. Failing to establish patient and public engagement and involvement (PPIE) can limit AI’s impact.

**Objective:**

This study aims to (1) understand patients’ and the public’s perceived benefits and challenges for AI and (2) clarify how to best conduct PPIE in projects on translating AI into clinical practice, given public perceptions of AI.

**Methods:**

We conducted this qualitative PPIE focus-group consultation in the United Kingdom. A total of 17 public collaborators representing 7 National Institute of Health and Care Research Applied Research Collaborations across England participated in 1 of 3 web-based semistructured focus group discussions. We explored public collaborators’ understandings, experiences, and perceptions of AI applications in health care. Transcripts were coanalyzed iteratively with 2 public coauthors using thematic analysis.

**Results:**

We identified 3 primary deductive themes with 7 corresponding inductive subthemes. Primary theme 1, advantages of implementing AI in health care, had 2 subthemes: system improvements and improve quality of patient care and shared decision-making. Primary theme 2, challenges of implementing AI in health care, had 3 subthemes: challenges with security, bias, and access; public misunderstanding of AI; and lack of human touch in care and decision-making. Primary theme 3, recommendations on PPIE for AI in health care, had 2 subthemes: experience, empowerment, and raising awareness; and acknowledging and supporting diversity in PPIE.

**Conclusions:**

Patients and the public can bring unique perspectives on the development, implementation, and embedding of AI in health care. Early PPIE is therefore crucial not only to safeguard patients but also to increase the chances of acceptance of AI by the public and the impact AI can make in terms of outcomes.

## Introduction

### Translating Artificial Intelligence Into Clinical Practice

Artificial intelligence (AI) is widely considered to be the new technical advancement capable of a large-scale modernization of health care. Current AI applications are advanced in their analytical power and precision to optimize health care logistics and support health care professionals in clinical decision-making [1]. AI applications can therefore play a vital role in improving the speed and accuracy of triage, diagnosis, and the quality of health care provided. Driven by AI’s funding opportunities to accelerate the use of AI in health care, manufacturers have developed a plethora of health care-related AI applications, yet the implementation and embedding of AI applications into health care currently face challenges that limit these applications’ success in clinical practice [2,3]. For example, van de Sande and colleagues [4] showed that more than 90% of AI prediction models developed for use in the intensive care unit remain within the prototyping environment, and very few make it to clinical practice. Key implementation challenges include issues around data sharing and privacy, transparency of algorithms, interoperability across multiple platforms, and concerns for patient safety [2].

Before a nationwide partnership of UK researchers on AI implementation in health care, we conducted a series of patient and public involvement and engagement (PPIE) consultations to understand public perceptions of AI in clinical practice at the earliest possible opportunity.

### PPIE Consultations

A primary challenge for AI applications is that their use of patient data will change existing relationships among patients, health care staff, clinicians, and health care systems [5]. Consequently, it is essential that patients and the wider public are involved in the design, development, implementation, and embedding of AI applications in health care. “PPIE” in the United Kingdom transforms research into a collaborative process, addressing and supporting the public’s needs [6,7], much like “patient-centered research” in the United States [8]. PPIE uses various types of qualitative data collection and project management methods to support social interaction–based collaboration between researchers and members of the public [6-10]. It allows patients and members of the public who will likely be affected by research outcomes to be involved in the research process, shaping research questions, collecting data, disseminating results, etc [6-10]. Building on the National Institute of Health and Care Research’s (NIHR) guidelines, we conducted a series of PPIE workshops or “consultations” with “public collaborators,” members of the public who discuss and provide feedback on research with researchers [6,9,10].

PPIE can prevent inaccurate information or misconceptions about AI applications from spreading while also yielding AI applications that reflect the public’s understanding, needs, and trust [11]. AI developers and purchasers who fail to establish PPIE in the development, implementation, and embedding of AI applications risk harming patients, losing public funds, damaging reputations, and having limited impact [12].

Presently, PPIE in AI research has focused on the public’s willingness to share medical data to enable “training” of AI applications [5]. Correspondingly, research literature reflects limited insights into public perceptions of the challenges and opportunities of AI in health care and how members of the public can and desire to be involved in the development, implementation, and embedding of AI [5]. A recent scoping review on PPIE and AI-assisted mental health care echoes this, arguing that additional research is required to understand which PPIE methods are most useful across differing public lived experiences and how to best apply them throughout AI development processes, especially given ethical issues surrounding AI and patient data and clinician and patient support for AI [13]. Interestingly, gray literature differs from this and instead focuses on the public’s broad and specific apprehensions around AI and research, some of which include algorithm bias, health inequalities, and damaging clinician-patient relationships [14-16].

### Aims

We conducted this consultation to begin a process of understanding and supporting connections between patients, the public, and academic communities, given that patients and the public are becoming more aware of AI applications’ roles and that researchers across Applied Research Collaborations (ARCs) are increasingly interested in AI in implementation [12].

This consultation aimed to (1) understand patients’ and the public’s perceived benefits and challenges for AI and (2) clarify how to best conduct PPIE in projects on translating AI into clinical practice given public perceptions of AI.

## Methods

### Setting

This PPIE consultation was conducted in the United Kingdom, where the NIHR funds and supports applied health research and research on the implementation of health and care evidence into clinical practice [17]. The NIHR achieves this through 15 regional interdisciplinary research entities, ARCs, covering all of England [18]. ARCs include institutional affiliations such as health care providers, universities, charities, and local government authorities and aim to improve patient and public outcomes and the quality, delivery, and efficiency of health care services.

### Public Collaborators and Recruitment

ARC North Thames (ARC NT) circulated an invitation for participation in the PPIE consultation through email to the 15 ARCs’ PPIE teams, requesting to share with their PPIE and public advisor networks. As each ARC handles PPIE differently and possesses a unique public advisor network, recruitment varied across the nation. Public collaborators were invited to respond to the invitation with their interest and answers to two questions: (1) Why would you like to join the AI interest group? (2) What skills and experience you would bring to the group?

Once the organizers of the PPIE consultation (WL and ARC NT core team staff) received no new notifications of interest (roughly 3 weeks’ time), they reviewed all responses (n=26) and assembled a convenience sample based on public collaborators’ stated interest. A total of 3 respondents did not give additional details, nor did they answer the questions included. The organizers followed up with them but received no responses. All individuals who wrote with interest and provided responses to the questions (n=23) were invited to participate, of whom 17 participated. The 6 respondents who abstained gave no reason for not participating. Per NIHR guidelines, participants were remunerated at £25/hour (US $31) and another £5/meeting (US $6) to support the cost of internet [19]. Overall, 4 of the 17 participants were known to WL before the PPIE consultation through ARC NT PPIE activities. WL is the PPIE lead at ARC NT and regularly conducts similar PPIE consultations and activities. Further communications were conducted through email.

### PPIE Consultation Structure

We used a combination of public involvement and focus group (FG) methodologies to reach public collaborators’ understandings, experiences, and perceptions of AI applications in health care [20]. We designed a semistructured FG topic guide incorporating deductive and open-ended inductive questions to allow ideas to emerge from the discussion while providing flexibility to probe topics and clarify understandings (Multimedia Appendix 1) [21]. A total of 17 public collaborators were distributed across 3 consultations to support a variety of experiences and perspectives in each group. This allowed us to explore public collaborators’ thoughts with differing questions and contexts, allowing for the illumination of different “…concepts, meanings, and explanations” [22].

Groups were cofacilitated by WL and CS with 5-7 public collaborators each. One more note-taking facilitator from the ARC NT staff was present for 2 sessions. All sessions were held through Zoom (Zoom Video Communications) to logistically support public collaborators’ participation from across England. At the start of each session, public collaborators verbally consented to contribute to the session’s recording. We ensured all had the greatest opportunity to participate by encouraging chat communications, reading chats aloud, supporting participants to deactivate video as needed, and using breakout groups to minimize pressure to speak.

Consultations were structured as follows:

1. general introduction and explanation of ground rules (10 minutes);
2. semistructured breakout group discussion (40 minutes);
3. presentation by CS defining and explaining implementation science and the implementation of AI into clinical practice (10 minutes); and
4. semistructured collective group discussion with all public collaborators, building on breakout discussions and CS’s presentation (50 minutes).

We treated breakout and collective group discussions as FGs to take advantage of the social context of discussing ideas in a group format. This allows public collaborators to “reflect and refine” their ideas, enriching their insights into their perspectives [23]. Breakout groups were held before CS’s specific project presentation to hear more “raw” perceptions of AI, unaffected by official explanations, and later enriched in the group discussion following the presentation.

### Analysis

Consultations were recorded and closed captioned with Zoom’s features, then used to create raw transcripts that WL edited, cleaned, and pseudonymized. All authors reviewed transcripts, discussed discrepancies, and WL made clarifying edits.

For the thematic analysis conducted in the study, data saturation was deemed of less importance than information power, requiring an information-dense sample rather than a large sample [24]. Analytic “codes” illuminated “themes” or descriptive narratives that summarize key discussions across all FGs [25].

All authors participated in an iterative qualitative coding process. SS and JH, 2 public collaborators in the FGs, also coded as public coauthors and coanalysts to assure the integrity and validity of the analysis. The analysis was done in 3 iterations, as shown in Textbox 1.

We use the Consolidated Criteria for Reporting Qualitative Research (COREQ) [27] to elaborate on our methodology (Multimedia Appendix 2).

**The structure and order of our coding and analysis process. Author attributions are included to illustrate collaboration.**

- First iteration
  WL, CS, SS, and JH reviewed transcripts, highlighted key passages, and created an initial list of in vivo codes [20,22].
  MS, WL, and CS reviewed transcripts with Dedoose qualitative coding software; SS and JH used Microsoft Word (Microsoft Corp) to code data from a single session different from the one they attended.
  All authors met to discuss data and codes and cocreate a single coding framework.
  JH’s and SS’s coded Word documents were uploaded to Dedoose for further review and coding. This was updated with each iteration.
- Second iteration
  SS and JH recoded two-thirds of the total data using this framework to assure interrater reliability.
  MS, SS, and JH discussed any discrepancies and created the final coding framework.
- Third iteration
  SS and JH used the final framework to finish coding.
  MS and WL reviewed coded data, identifying “themes” or descriptive narratives illuminated by coding that summarized key discussions across all focus groups [25,26].
  Written themes were reviewed and approved by all authors.

### Ethical Considerations

Research ethics approval for PPIE consultations is not required, per the NIHR [10]. However, as stated above, collaborators consented to FG participation.

## Results

### Public Collaborators

This group of public collaborators participating in the consultation is not intended to be representative of the public in general but rather represents a subset interested in collaborating on research. They offer layers of perspective based on their experiences, some of which included those of patients, caregivers (“carers” in the United Kingdom), research public advisors and collaborators, health or research volunteers, health care professionals, and in-patient participation networks. In line with the information density principle described in the Methods section, most public collaborators represented 2 or more roles from which they could contribute to the study. We did not collect data specifically on demographics in a standardized way, as we wanted to leave participants open to speak about their identities, backgrounds, and subjectivities as they felt led. Some participants offered up this information during the FGs and their expressions of interest in participating. Additional details on their interests and backgrounds can be found in Multimedia Appendix 3.

Public collaborators varied in their experience with and knowledge of AI in health care. These included (1) some with no preexisting knowledge of AI; (2) some with preexisting knowledge through AI research and digital innovation in health care, panels awarding funding for AI projects in health care, and research or implementation projects of small-scale AI projects in health care; and (3) 1 participant with experience constructing AI scripts.

Public collaborators represented 6 different NIHR ARCs (North Thames, North West Coast, South London, West Midlands, Yorkshire and the Humber, and South West Peninsula).

### Themes

Through iterative analysis, we identified 3 primary deductive themes, with 7 corresponding inductive subthemes (Table 1).

**Table 1:** Overall theme’s structure.

Themes and subthemes		Key points by subtheme
**1. Advantages of implementing AI^a^ in health care**		
	1.1 System improvements	AI could help improve the processing, analysis, and interpretation of the vast amounts of health care data currently collected from patients
	1.2 Improve quality of patient care and shared decision-making	Patients would like to receive AI-related patient information from clinicians
**2. Challenges of implementing AI in health care**		
	2.1 Challenges with security, bias, and access	AI could be used for things harmful to patients
	2.2 Public misunderstanding of AI	Public understandings or conceptualizations of AI can be based on misunderstandings or inaccuracies
	2.3 Lack of human touch in care and decision-making	The development and implementation of AI consider the volume of processing patient appointments and services over specific patient needs
**3. Recommendations on PPIE^b^ for AI in health care**		
	3.1 Experience, empowerment, and raising awareness	PPIE in the development and implementation of AI in health care will be advantageous to AI functionality, AI effectiveness, and the public’s sense of ownership of AI
	3.2 Acknowledging and supporting diversity in PPIE	There is a need to recognize how diversity, in the form of “ethnic background,” “cultural perceptions,” and “protective characteristics,” affects public perceptions and experiences of AI

^a^AI: artificial intelligence.

^b^PPIE: patient and public engagement and involvement.

### Primary Theme 1: Advantages of Implementing AI in Health Care

Public collaborators’ perceived advantages of implementing AI in health care focused on subthemes 1.1: system improvements and 1.2: improve quality of patient care and shared decision-making.

#### Subtheme 1.1: System Improvements

First, public collaborators discussed how AI could help improve the processing, analysis, and interpretation of the vast amounts of health care data currently collected from patients.

In the future, the researchers, when they have large data, AI will help to accurately analyze them.

FG1

Public collaborators described that the data analysis done by AI could help to “revolutionize health much, much better and quickly” [FG2], for example, by uncovering geographical differences in disease prevalence and by identifying differences in performance between health care centers. Public collaborators thought AI could perform these tasks more efficiently and cheaply, compared to humans, and could potentially reduce the workforce burden experienced in the National Health Service (NHS). Several public collaborators also expressed the hope that AI could benefit communication between health care professionals and patients. Patient and caregiver public collaborators discussed the current lack of communication between departments and specialties within and between hospitals. This was considered detrimental to patient care, especially for the more complex cases requiring multidisciplinary health care. These public collaborators expressed hope that AI could aid this communication by making meetings’ information sharing more accessible.

I think, as a carer, sometimes it’s very frustrating when different departments within one hospital do not communicate with each other, and if AI can improve that part, then it’ll make life a lot easier for carers as well as the patient.

FG2

#### Subtheme 1.2: Improve Quality of Patient Care and Shared Decision-Making

Second, public collaborators discussed how AI could potentially benefit patients and caregivers directly, for example, through improvements in the care and services provided to patients. Public collaborators mentioned that they thought AI could reduce waiting times and improve the quality of the information provided to the patient during consultations (eg, a more accurate prognosis), thereby facilitating patient empowerment.

If the information used by the doctors that they have gathered from AI is shared with the patient…it’s very helpful. The more information, the more knowledgeable I will be...If I’m given information about the future of my, [condition] then …the more information I get, the more empowered I say I would feel.

FG2

Furthermore, public collaborators thought that AI could play a crucial role in improving care by reducing the number of human errors made in diagnosis and treatment, facilitating early detection of disease, and improving the development and quality of treatments and medications.

AI can even detect things before […] a human can.

FG3

There are a lot of facets to AI […]. AI can be used for detection…monitoring…management…decision making…as a carer, I think there is a lot of elements to AI, which I don’t think healthcare providers are using enough.

FG2

Public collaborators also considered AI an opportunity to reduce bias in health care.

Nowadays, in the feel of health inequality and so on, I feel sometimes AI perhaps can be a fairer instrument.

FG3

Some public collaborators perceived AI as not being influenced by a patient’s skin color or politics, increasing the chance that patients from ethnic minorities would be helped more fairly.

### Primary Theme 2: Challenges of Implementing AI in Health Care

Three main subthemes emerged regarding public collaborators’ perceived challenges of AI in health care, including subtheme 2.1: challenges with security, bias, and access; subtheme 2.2: public misunderstanding of AI; and subtheme 2.3: lack of human touch in care and decision-making.

#### Subtheme 2.1: Challenges With Security, Bias, and Access

Public collaborators found challenges of security, bias, and access around the data needed to train AIs and make them function. They felt AI could pose risks to personal data security, especially when data are handled by private companies, whom they considered to have less strict security than the NHS and higher chances of selling personal data for profit.

…where would the information be stored […]? Would it be with the NHS, which is more secure, or would it be with private companies who can then sell it on, make a profit, or if the private company goes bust, […] where does all the hard work […] go?

FG2

Contributors also debated who would be responsible and accountable for AI and the data they require, largely because they considered AI a new phenomenon with unclear rules on data sharing and management.

We are looking at technology which hasn’t got ruled in regulations…how are we gonna manage this, you know, in the future?

FG2

AIs were consequently perceived as prone to manipulation, again leading to their use for financial incentives.

Additionally, contributors expressed concern about AIs’ tendency to replicate human biases around race and identity by learning from data that already reflects such biases and a lack of diversity in developers. One compared this to similar issues with UK police AI applications:

It’s like with the police force, the facial recognition and AI […] picking up more Black people than the white population… we have to consider those kind of ethical questions.

FG2

Contributors emphasized a response of including people beyond populations in developing AIs to minimize bias, specifically by training the AI with data from various communities that consider health inequalities. One emphasized:

…we have to be careful of…when we code the programming for AI, that [it] isn’t just the white population.

FG2

Finally, access to AI technologies defined a portion of the discussion around challenges, including disability, literacy, age, and technology access:

…the system is set up for X, Y, and Z, and you can’t move outside the system or things can’t be adapted for people with special needs or disabilities, that then they can’t access things in a certain way, or the service can’t work to meet their needs.

FG3

…people [who] are aged and have less IT knowledge…how will they use it for themselves? Very low literacy also-how to make it possible for them?

FG1

Some contributors warned that AI, through challenges like access and bias, could increase inequality.

We need to think about how it’s going to affect everyone. I think we are running in terms of artificial intelligence and some people are going to get left behind.

FG1

#### Subtheme 2.2: Public Misunderstanding of AI

Public collaborators perceived that members of the public possess limited knowledge of AI, including misconceptions based on popular culture that can instill fear or confusion in the wider public:

When AI came out, I probably picked it up first from movies like Arnold Schwarzenegger. That represented to me that [it] is a fantasy world.

FG3

Public collaborators found the current presentation of AI in public contexts to be presented in abstract ways, with applications in daily life being scarce, unknown, or limited to health services like SMS text message notifications for appointments. Thus, this complicated contributor’s understanding of AI’s relevance to their lives. While some public collaborators demonstrated a robust understanding of AI, others were unclear, with one saying,

This artificial intelligence, I didn’t understand how they are using it.

FG1

Beyond misconceptions and limited knowledge, most public collaborators described a need to increase or cultivate patients’ understanding of the clinical roles of AI and how they work:

How AI will be made familiar for the community? How people will know [sic] about this? When you have different morbidities, many time [sic], you are more concerned about what will happen; ‘If I have this, my blood pressure will raise, my sugar will raise.’ How can we, with these machines, help people? How can we make it possible?

FG1

#### Subtheme 2.3: Lack of Human Touch in Care and Decision-Making

A final perceived challenge was that AI could threaten patients’ health needs. Public collaborators perceived that the development and implementation of AI consider the volume of processing patient appointments and services over specific patient needs.

…from my previous experience of technology potentially being implemented for patients, it’s not necessarily being for the patients' benefit. It’s been for a service perspective.

FG3

That’s when patients fall off the side. Because it has improved…the efficiency of the system. We can get x number of patient appointments back in quicker…. But actually, that wasn’t what the patient needed.

FG3

I fear that the AI might be putting people down more of a generic path and there might be…less ways of challenging it, if it’s decided by AI rather than a human.

FG3

One contributor described the AI clinical decision-making process as “binary,” restricting decisions to yes or no and excluding a “maybe” option.

This correspondingly led to a “lack of human touch” in health care, hurting relationships between patients and clinicians, and ceding decision-making power to AIs and away from the patient-clinician dyad.

I’m still a bit concerned about AI being used to decide what treatments and pathways patients might be eligible for…if someone’s a patient’s discussing their needs with a clinician, they’re bound to be able to bring out more…individual needs of that patient.

FG3

One contributor even described a theoretical scenario in which a patient felt a “loss of autonomy” caused by AIs taking over more decision-making in their care.

I think there’s always a fear that, ‘the machine will do everything on behalf of myself and on behalf of the doctor,’…that’s a fear of loss of autonomy, that, you are just going to be disregarded…I think it gets lost-the point that AI is only decision aid…

FG3

Furthermore, public collaborators feared that properly implemented AI could still add rigidity to health systems and processes. Perceived consequences of this included AIs limiting patients’ treatment options due to a need to fit into the digital system and less flexibility provided by human support.

I feel sometimes, patient safety could be endangered if you have, a very rigid, algorithm, that overlook [sic] some sometime very vital clues.

FG3

Finally, public collaborators discussed the maintenance of AI technologies as a challenge. They perceived that AIs would need continuous updates corresponding to new medical discoveries, technological advances, clinician needs, and patient needs.

### Primary Theme 3: Recommendations on PPIE for AI in Health Care

The 2 main subthemes emerged regarding public collaborators’ recommendations on PPIE for AI in health care, including subtheme 3.1: experience, empowerment, and raising awareness; and subtheme 3.2: acknowledging and supporting diversity in PPIE.

#### Subtheme 3.1 Experience, Empowerment, and Raising Awareness

Public collaborators thought the involvement of patients and the wider public in the development and implementation of AI in health care would be advantageous to the functionality and effectiveness of AI as well as to the sense of ownership of patients and the public.

You must involve patients and families and carers in that development and the design […]. Without that […] systems will be meaningless or less effective.

FG2

Collaborators saw public collaborators and patients as useful additions to AI projects to safeguard patient perspectives and guarantee the impact of AI projects: “The patient really views, they’re really crucial to know whether it’s actually improving patient care” [FG3]. Public collaborators elaborated that involvement should come from people with varying backgrounds:

You need different people who will want to contribute or who will want to share experience […]. People who will be sitting on the ethics and approval panel where they will look at how the systems are being […] delivered in a meaningful way.

FG2

An additional benefit of PPIE in the development and implementation of AI in health care was thought to be increased awareness of AI, thereby counteracting some of the misconceptions and fears that arise from a lack of knowledge.

Public collaborators mentioned that training the patients and the public could help to empower and educate them, which in turn will help to get “meaningful input in the meetings” [FG2].

#### Subtheme 3.2: Acknowledging and Supporting Diversity in PPIE

Collaborators’ recommendations for PPIE coalesced around the need to recognize how diversity, in the form of “ethnic background,” “cultural perceptions,” and “protective characteristics,” affects public perceptions and experiences of AI.

They emphasized several mechanisms to support and benefit from diversity in PPIE around AI. First, this included co-design at early and consistent stages of the project:

The best thing you can ever do is co-design all the messaging and the stuff that goes into the back end of the ethics applications…the richness, the creativity, the, you know, the resonance and that rapport building just happens so much more quickly when it’s been codesigned and written as a collaborative.

FG2

Second, it included balancing the numbers of researchers and public contributors while likewise using inclusive and accessible language.

[There’s a need for a] balance of academics and public’s contributors because…public contributors can feel intimidated…the use of the language they’re coming out with…which public contributors may not understand…it can be very off putting and therefore…people would think, ‘oh, well, I’m not gonna bother attending this. It was so boring…And half the time, I didn’t understand...’

FG2

Third, some collaborators focused on practical aspects, such as balancing the numbers of public contributors and professionals in meetings.

I’ve been in certain meetings where there is like 30, 25 to 30 professionals and only three or four public contributors, and that imbalance can impact on one’s ability, to contribute.

FG2

Fourth, 1 collaborator suggested even including clinical staff in the PPIE process to have a more ecumenical collaboration encompassing the practical translation of the AI projects:

I think it’s not just the patients. You would need…the inclusion of the clinicians, … the frontline staff, and the patients…the mixed group of people’s views in prioritizing that.

FG2

Fifth, some cited specific identities and groups that could be supported by having research and clinicians with similar ethnic makeup to public members:

…certain Black minority ethnic people. They feel that this is yet another white exercise for white people. I’ve got dark skin…, [and I might think], ‘Doesn’t apply to me. It’s not meant for me.’ But if you’ve got clinicians, leaders, researchers who have got their [same] background, they will appeal to a certain group…

FG3

One participant suggested that these recommendations take the form of an advisory group in which researchers consider their needs alongside public contributors’ varying abilities and availabilities.

…you will need sort of an advisory group of being able to bounce off ideas as a panel…lay out really, what are the levels that you would like people, depending on your program needs, so that we can then say, ‘yes, I’m happy to contribute at this level or that level.’

FG2

## Discussion

### Main Findings

This consultation explored public collaborators’ perceptions of the implementation of AI in health care, and it found 3 primary deductive themes with 7 corresponding inductive subthemes.

Primary theme 1: advantages of implementing AI in health care, and its 2 subthemes: system improvements and improve quality of patient care and shared decision-making.

Primary theme 2: challenges of implementing AI in health care, and its 3 subthemes: challenges with security, bias, and access; public misunderstanding of AI; and lack of human touch in care and decision-making.

Primary theme 3: recommendations on PPIE for AI in health care and its 2 subthemes: experience, empowerment, and raising awareness; and acknowledging and supporting diversity in PPIE.

Across these themes, public collaborators noted several advantages of AI in health care, such as improved quality and efficiency of health care systems, as well as better diagnosis and treatment for patients. Public collaborators also discussed ethical and regulatory challenges for the implementation of AI as well as the resistance the public and patients feel toward AI due to concerns over bias and access, data protection, and perceived threats AI poses to patient care. Overall, public collaborators felt that patients and the public need to play a role in AI development and implementation to safeguard patient care and maximize AI’s efficacy in practice.

### Comparison With Previous Work

This consultation’s contribution lies in (1) consolidating recent findings across various stakeholders and fields of research (data, AI, co-design, PPIE, and qualitative research); (2) connecting key themes represented in patients to those represented in other stakeholders; and (3) connecting gray and research literature.

The current research literature on the implementation of AI focuses largely on health care professionals’ perceptions instead of those of patients and the public [28-32]. Interestingly, these professionals’ perceptions overlap with those of the public in our consultations. Like our “advantages” theme, health care professionals in the studies of Laï et al [31] and Choudhury and Asan [29], for example, found AI can save doctors’ time, reduce medical errors, and improve diagnosis and treatment for patients. Interestingly, they encountered similar concerns around AI as our challenge subthemes; for example, data accountability and security, ethical dilemmas, limited understanding of AI, and detrimental impacts on physician-patient relationships could be key barriers to the successful implementation of AI [28,29]. Several of these “concerns” were echoed in public involvement literature focusing on patient perceptions of health data use, which often touched on AI in health care [33,34]. These included health data used for profit, discrimination bias, “loss” of the patient, and weakening of the patient-clinician relationship [33,34]. Recent gray literature reports from the NHS AI Lab and NHS AI Lab awardees further illustrate extremely similar themes as our findings, including concerns about algorithm bias, harming clinician-patient relationships, health inequalities [13-15], and general fear and misunderstandings [16]. What is more significant is that our consultation not only reiterates several of these concerns and hopes but does so from a combination of researcher and public perspectives.

This consultation shows that public involvement is both desired by the public and key to the development and implementation of AI in health care. Successful implementation and adoption of AI in health care will require consideration of human factors beyond AI’s predictive power and technological capabilities [29]. Patients and the public can serve as collaborators with AI in health care, either through public involvement, coproduction, co-design, or any similar methodology, and can thus shape a form of AI that addresses these concerns, a position shared by other projects [13-16,33-35]. In facilitating relevant PPIE activities, researchers need to be especially careful of the themes identified by this consultation: experience, empowerment, raising awareness, and acknowledging and supporting diversity in PPIE. PPIE can assist in reducing the so-called development-to-implementation gap of AI, referring to the mismatch between the goals for which AI is developed by manufacturers and the needs of practitioners and patients in practice [36,37]. Patients and the public bring unique knowledge of what they need when undergoing health care and how AI can help fulfill these needs.

Our consultation suggests that PPIE can lead to more awareness of AI among patients and the public, but it also inspires a sense of ownership of one’s treatment. Not only can this support resolving some of the misconceptions and fears that the general population has toward AI, but it can also lead to increased trust in and acceptance of AI by patients and the public. In a time where personalized medicine and shared decision-making are at the forefront of accepted good clinical practice, engaging patients in the development and application of AI is just as important as engaging patients in their usual health care trajectories.

This study follows a few recommendations for policy and practice. Early engagement of patients and the public in the AI development and implementation journey as well as in designing policy on AI implementation is important. PPIE should represent people with varying backgrounds, such as different ethnic backgrounds, various health conditions and disabilities, various ages, and those with varying AI knowledge and expertise, as these determinants can impact the needs and therefore the usability of various AI applications. Educating patients and the public can aid in achieving successful PPIE by making sure that everyone can contribute and by resolving some of the misconceptions about AI [32].

### Strengths and Limitations

This study not only engaged public collaborators as PPIE consultants but also involved 2 public collaborators in the analysis and interpretation of the data presented in this paper. It was also then reviewed by 3 additional public collaborators. These approaches strengthened these results by guaranteeing that the perspectives of public collaborators were respected and represented in the results. The size of the consultation can be considered a limitation of the study; however, given the depth of the data, it is unlikely that a larger sample would have changed the results substantially, especially given connections across other literature. Notably, similar PPIE consultations have had similar sizes [30]. Future research should expand beyond members of the public accustomed to PPIE activities, such as those included in this consultation.

### Conclusion

We conducted a patient and public involvement consultation with individuals connected to NIHR ARCs from across England to center their perceptions on AI and the translation of AI into clinical practice in anticipation of a nationwide, cross-ARC research collaboration. This consultation supported similar studies and PPIE work conducted with different populations on AI and data sharing, illustrating the strength of these themes and their relevance across different stakeholders. This cements the need for AI in clinical practice to be co-designed with a diverse group of patients and members of the public.

## Appendix

Multimedia Appendix 1 The topic guide we used to guide focus groups.

Multimedia Appendix 2 Consolidated criteria for reporting qualitative research checklist.

Multimedia Appendix 3 Expressions of interest, providing additional background information on participants.

## Abbreviations

AI: artificial intelligence
ARC: Applied Research Collaboration
ARC NT: Applied Research Collaboration, North Thames
COREQ: Consolidated Criteria for Reporting Qualitative Research
FG: focus group
NHS: National Health Service
NIHR: National Institute of Health and Care Research
PPIE: patient and public involvement and engagement

## Data Availability

The data sets generated during or analyzed during this study are available from the corresponding author on reasonable request.
