# Supplementary material for: Centering Public Perceptions on Translating AI Into Clinical Practice: Patient and Public Involvement and Engagement Consultation Focus Group Study
Source: J Med Internet Res. 2023 Sep 26;25:e49303. doi: 10.2196/49303 (PMC10565616; doi:10.2196/49303)
Supplement: Multimedia Appendix 1 [file jmir_v25i1e49303_app1.docx]

## Supplementary material 1: semi-structured focus group topic guide

| Breakout groups | How did you become interested in AI? |
| --- | --- |
|  | Probes – If not, why not? |
|  | What concerns do you have about AI in healthcare? |
|  | Probes – What is it getting wrong and right? What else could it be doing? |
|  | What are your anxieties about AI in general? About health care and AI? |
|  | What opportunities do you think it holds? |
|  | How do you understand AI in the clinical setting? |
|  | Probes – What do you know about it? Have you ever experienced this before? |
| Group discussion | Now that you’ve heard more details on the project, what are your thoughts and impressions of it? |
|  | Does it change your thoughts on AI and AI in healthcare? |
|  | Probes – Are your hopes and concerns the same? |
|  | How should the public be involved in the project? |
|  | Probes – How would you want to be involved in this? |
|  | What should we absolutely keep in mind? |
|  | How can we go about this project ethically and supportively? |
