# Supplementary material for: Centering Public Perceptions on Translating AI Into Clinical Practice: Patient and Public Involvement and Engagement Consultation Focus Group Study
Source: J Med Internet Res. 2023 Sep 26;25:e49303. doi: 10.2196/49303 (PMC10565616; doi:10.2196/49303)
Supplement: Multimedia Appendix 2 [file jmir_v25i1e49303_app2.pdf]

Supplementary material 2: COREQ Details

COREQ (COnsolidated criteria for REporting Qualitative research) Checklist

Topic	Item No.	Guide Questions/Description	Reported on Page No.
**Domain 1: Research team and reflexivity**			
Personal characteristics			
Interviewer/facilitator	1	Which author/s conducted the interview or focus group?	2, 3
Credentials	2	What were the researcher's credentials? E.g., PhD, MD	1
Occupation	3	What was their occupation at the time of the study?	1
Gender	4	Was the researcher male or female?	n/a
Experience and training	5	What experience or training did the researcher have?	2
Relationship with participants			
Relationship established	6	Was a relationship established prior to study commencement?	2
Participant knowledge of the interviewer	7	What did the participants know about the researcher? e.g., personal goals, reasons for doing the research	2, 3
Interviewer characteristics	8	What characteristics were reported about the interviewer/facilitator? e.g., Bias, assumptions, reasons and interests in the research topic	2, 3
**Domain 2: Study design**			
Theoretical framework			
Methodological orientation and Theory	9	What methodological orientation was stated to underpin the study? e.g., grounded theory, discourse analysis, ethnography, phenomenology, content analysis	2, 3
Participant selection			
Sampling	10	How were participants selected? e.g., purposive, convenience, consecutive, snowball	2
Method of approach	11	How were participants approached? e.g., face-to-face, telephone, mail, email	2
Sample size	12	How many participants were in the study?	2
Non-participation	13	How many people refused to participate or dropped out? Reasons?	2

Setting			
Setting of data collection	14	Where was the data collected? e.g., home, clinic, workplace	2, 3
Presence of non-participants	15	Was anyone else present besides the participants and researchers?	2
Description of sample	16	What are the important characteristics of the sample? e.g., demographic data, date	4, suppl. 3
Data collection			
Interview guide	17	Were questions, prompts, guides provided by the authors? Was it pilot tested?	2, 3
Repeat interviews	18	Were repeat inter views carried out? If yes, how many?	2, 3
Audio/visual recording	19	Did the research use audio or visual recording to collect the data?	2, 3
Field notes	20	Were field notes made during and/or after the interview or focus group?	2, 3
Duration	21	What was the duration of the inter views or focus group?	2, 3
Data saturation	22	Was data saturation discussed?	3
Transcripts returned	23	Were transcripts returned to participants for comment and/or correction?	3
**Domain 3: analysis and findings**			
Data analysis			
Number of data coders	24	How many data coders coded the data?	3
Description of the coding tree	25	Did authors provide a description of the coding tree?	N/A
Derivation of themes	26	Were themes identified in advance or derived from the data?	3, 4
Software	27	What software, if applicable, was used to manage the data?	4
Participant checking	28	Did participants provide feedback on the findings?	3, 4, 9
Reporting			
Quotations presented	29	Were participant quotations presented to illustrate the themes/findings? Was each quotation identified? e.g., participant number	5-8

Data and findings consistent	30	Was there consistency between the data presented and the findings?	5-8
Clarity of major themes	31	Were major themes clearly presented in the findings?	5-8
Clarity of minor themes	32	Is there a description of diverse cases or discussion of minor themes?	5-8
