# Supplementary material for: Centering Public Perceptions on Translating AI Into Clinical Practice: Patient and Public Involvement and Engagement Consultation Focus Group Study
Source: J Med Internet Res. 2023 Sep 26;25:e49303. doi: 10.2196/49303 (PMC10565616; doi:10.2196/49303)
Supplement: Multimedia Appendix 3 [file jmir_v25i1e49303_app3.docx]

## Supplementary Material 3 – Expressions of interest, providing additional background information on participants

Focus group participants consented to include this information and provided edits to it where they desired and felt appropriate.

| **Statement** |
| --- |
| I have a keen interest in research and have over ten years’ experience in advising on research as a lay advisor.  I have a number of long-term health conditions, and the community work I do supporting others and their carers gives me a wide ranging insight into the needs of those using health and social care services and as my work includes projects across the country my networks are large and diverse and I have a lot of experience using all of this to inform what I contribute.  I sit on the lay board of London IVD as well as the co-development panel of NHS digital.  These and other projects give me a wide-ranging interest in AI, and I am always keen to get involved in new projects and continue to develop my knowledge and experience as well as use all I can bring to the table to try and ensure that the care and treatment that all who have health issues need becomes the best it can be. |
| I am a PPIE contributor and would be interested in participating in your research project.  Why you would like to join the AI interest group? I am really interested in finding out how AI can be translated into clinical practice but am mindful that this could be implemented really well in ways that patients find beneficial and are comfortable with, but I also have reservations that it must be implemented in a way that is acceptable to all patients and its vital that AI demonstrates more than just financial benefits. It also needs to be something that staff and patients are comfortable and happy using.  What skills and experience you would bring to the group? I have been involved in a number of projects in health and social care as a patient and public contributor as well as a co-applicant on a research study and national healthcare guidelines. I have personal and professional experience working with people from diverse communities and with disabilities.  I feel my background experiences give me insight into a range of people's perspectives and views on new technology and the challenges and potential benefits they may identify from the changes. I have also successfully implemented changes into practice and seen challenges implementing new guidance. Please don't hesitate to contact me should you wish to have any further information. |
| I am very much interested in translating Artificial Intelligence into practice.  My background was in Engineering and IT before I became a PPI volunteer. I can offer my practical knowledge in this area.  AI will help both clinicians and patients in eradicating mistakes made due to human errors and speed up many processes.  I was involved a few years ago with Google/NHS as a PPI on their research which was called Deepmind, which looked at the diagnosis of eye diseases. I believe the NHS has now withdrawn their involvement.  I believe in the future AI will be used in remote diagnosis by clinicians. As more and more data is gathered by the NHS which will be used by the researchers for health improvements, AI will be a useful tool for them.  I was recently involved in reviewing a few projects proposed by private companies via NIHR. These companies were involved in using AI for their projects in health care. |
| I would like to be involved in the AI interest group because I am interested in AI in the NHS, and I would learn a lot. I would be interested to see how it functions and the meetings will be at the cutting edge of NHS work and research.  I am very interested in how AI will benefit patients. The NHS is looking at Digital Health options and AI because patients who need help will be diagnosed quicker and the systems will save time. Digital Health is about the future and AI information can be used by doctors to recommend further tests or treatments. New Digital Health applications can be linked to the AI work in the NHS and in research in the future.  For these reasons, I am interested in being part of developing the 'position paper' that discusses the key research that is involved in translating AI into practice.  I have a BA and the Cambridge CELTA and DELTA in English teaching, so I have the ability to read documents attached to this work. I also have 10 years' experience of being the co-chair of the Service User Research Forum at UCL and 4 years' experience of the RAP as a member at the ARC North Thames. I have discussed many research proposals and presentations in these roles. I am a good communicator and listener and would find developing the 'position paper' very interesting. |
| I am a public adviser for NIHR ARC Northwest Coast Team and also hold a Computing Degree. I have always wanted to be involved in the care and health informatics sector of NIHR ARC research.  Artificial Intelligence (A.I.) fascinates me a great deal as computer technology is increasing at a rapid pace, it is important that we use this technology to improve our national health service in the future. If this means using A.I. to early detect the signs of pancreatic cancer or other cancers that are difficult to diagnose in the early stages or even to prevent and predict a future pandemic like COVID occurring again by using A.I. big data.  When it comes to skills and experience which I have developed whilst being a public adviser for NIHR ARC NWC, my qualitative research skills have been developed a great deal. I am very passionate about qualitative research methodology such as thematically analysing interview transcripts finding key coding themes present within all the transcripts. I personally feel that you can retrieve more valuable rich meaningful data from qualitative research compared to quantitative which is just statistical graphs and charts which doesn`t give you a comprehensive analysis of the data. I do believe that A.I. tools can be used in the future to collect qualitative health data as nowadays people are using sophisticated A.I tools like smart watches which track a person’s body intake, heart rate, etc. The big data collected from these devices can then be qualitatively analysed to predict and prevent say the likeliness of residents living in certain regions in the United Kingdom being more prone to developing mental health problems, Cancer, or any other health issues. |
| I would be happy to participate in the Zoom in March regarding AI in health and care settings however have no prior experience on the subject. |
| Why you would like to join the AI interest group: The opportunity to have an input in Translating Artificial Intelligence (AI) into practice in health and care settings and making an impact by supporting the group is highly motivating.  What skills and experience you would bring to the group: I believe I can bring a unique and diverse perspective and make a significant impact on the research projects based on my professional background and more recently as PPI member.  For my most of my life, I have been blind as a result of suffering from Retinal condition.  Since retiring from BP several years ago, where I held various senior leadership posts in the areas of procurement and IT, I have been pursuing my passion in improving health care and in raising awareness and promoting the capabilities of disabled people.  More recently I was diagnosed with Myasthenia Gravis and CIDP, and, as such I have developed a deeper understanding of both the NHS and private sectors in both general and specific health needs. I believe the use of AI is capable of creating, transforming and improving many facets of human life.  It also plays a significant role in healthcare.  My experience in managing numerous business projects will help me to make a positive contribution – from inception to implementation and beyond...  For example, better user experience. Implementing AI in various industries involves applying intelligence to machines and technology used regularly by humans as AI does not work in isolation from devices. A popular example would be virtual assistants like Google Assistant which improves an existing product with enhanced features which benefit the user.  Based on my experience as PPI member over the past several years. The opportunity to provide input on a range of service design, delivery and research projects allowed me to develop a deeper understanding of healthcare issues and I have been able to provide increasing amounts of advice, insights and scrutiny to ensure successful outcomes.  Furthermore, I believe involving the public, patients and carers (PPI) early in the development of an innovation, in the evaluation of innovations and service design /improvements makes sense for a multitude of reasons – ranging from the practical to the ethical. Early active involvement and engaging the PPI at the right time during the research stage ensures buy in and promotes awareness. Subsequent implementation phases become smoother and less of an issue to manage in transition.  In short, PPI -focused research simply cannot be delivered without the involvement of the PPI. No matter how complicated the research, or how brilliant the researcher, the PPI always offers unique, invaluable and first-hand insights. Their feedback when designing, implementing and evaluating research invariably makes studies more effective, more credible, and more interesting and often more cost efficient as well. |
| I am Carer for a family member suffering from long-term multiple conditions. I am responsible for all self-manage medical, health and social care needs.  I am from Black, Asian, and Minority Ethnic (BAME) background living in an area with large health, cultural and socio-economic diverse communities. |
| As a person living with a number of health conditions, I am interested in the smart use of health data in learning about best practice for my conditions and also other conditions with potential promise for wider benefits for the public at large.  It seems to me that much can be learned about the connections and patterns in vast amounts of health and care data.  These discoveries can lead to new clinical insights which can help us to better prevent and treat diseases and identify people who are at risk of developing illnesses.  At population level, we could also find out where health inequalities may lie and take appropriate measures to address them.  I am keen to share patient and public's perspective in shaping these discussions to ensure the products will be important and relevant to them.  Over recent years I have had far and wide contact with patients and public, listening to their health issues of concern, such as through interviews in Care Quality Commission inspections to hospitals and general practices, primary care patient interest groups, HealthWatch citizen focus groups, and various on-line patient forums.  My dialogues also included under-served groups like certain minority ethnic groups. |
| I'm writing to express an interest in joining the AI Community. I'm an experienced patient representative with a background in health research and science. I'm really interested in new technology. I am a Lay Member of the Leeds Surgical MedTech, NIHR Imperial College Invitro Diagnostics Board, and the MHRA Interim Devices Working Group. I have experience of grant funding committee experience including RfPB, HTA, and the NHS SBRI Healthcare panel. I also sit on various committees with Pharmacy Research UK, the British Heart Foundation, and GOSH. I'm an autistic disabled adult living with long-term health conditions and am also a carer for someone who has autism. |
| I think AI could be useful for people who have mental health issues to overcome phobias which could be learned behaviour or trauma from childhood. I would like to be on the panel and give the team advice where best it can be used, not just NHS settings, but also in community groups and third sector organisations.  I have personally used an AI app called Youper, which has helped me with my anxiety to think differently in certain situations, and I think AI could help many people recover from their illness or reduce their stress.  I think AI could be another tool for mental health organisations to help aid the service user in their recovery and be a form of cognitive behavioural therapy and NHS do not just rely on medicines alone.  I am an ARC member and contributor to West Midlands ARC, also a member of various Mental Health organizations around the West Midlands, which help promote and empower service user involvement and participation. |
| The reason I would like to join the AI interest group is due to the following.  I have sensory disability and am also a carer to a close family member who has complex physical and mental health needs.  I have been an active lay member of North Thames ARC Research Advisory Panel for several years, member of UCL Centre for Co-Production in Health Research, since 2020 a selected member on the monthly ARC Population Health and Social Care theme meetings and am also involved in a Psychology project improving psychological therapies.   In 2018, as a patient of Moorfields Eye Hospital, I was invited to participate in a ophthalmology patient and public focus group looking at developing AI technology for diagnosis and referral in retinal optical coherence tomography.  I thoroughly enjoyed contributing to this project and also listening to other patients’ views.  I am keen to learn how AI could help health staff reach a swift diagnosis, save time and benefit patients.  I have an understanding, experience and involvement in research from my membership of the Research Advisory Panel of North Thames ARC. Being a service user myself and as a carer, I have an understanding and experience of using health and social care services. I believe that I have the capacity to contribute to a discussion and am open and respectful of other people’s views and opinions and try to be non-judgemental. |
| I am undertaking a PhD at York St John University looking at digital clinical safety focused on AI/IoT technology in social care. In particular, I am interested in how occupational therapists (I am one, of course!) communicate around digital clinical safety.    In my day job, I am a Clinical Safety Officer and work for companies supporting their compliance with the DCB0129/0160 NHS England safety standards, in particular within the bounds of research and innovation projects…amongst other things. I lead projects and programmes across health and care relating to the design and deployment of tech. I do a lot of co-design work with the public, clinicians and researchers.    I have worked across health and social care, research and innovation and in commissioning, applying my clinical knowledge and experience to improve services and people using services or their carers experiences. |
| I live in Lancashire. I am working as Public Adviser ARC NWC almost one year. I have 25 years’ experience of development sector in Pakistan before coming to UK. I worked in health projects mainly, where my last job was with World Health Organization as a provincial communication officer for Polio Eradication program in Baluchistan province/ Pakistan.  I am interested in opportunity of Translating Artificial Intelligence (AI) into practice in health and care settings research. I worked with different vulnerable groups specially women, adolescents and children. As person from the minorities group south Asian, Pashtoon ethnicity, that can bring a good exposure to the group. |
| In terms of experience and interest in AI, I have taken part in various AI projects in both research and commercial practice where I have supported funding allocation decisions, service user involvement and considerations from both patient/carer point of view. I have an interest in the applications of AI for rare diseases, complex multimorbidity and neurology areas but a fairly good understanding of both medicines R&D and health research through patient expert training. I freelance as a grants writer primarily helping SMEs get funding for innovation and have 20+ years professional experience in stakeholder engagement, project management, EU grants, tech transfer and start-ups. I have also worked as a research engagement and knowledge exchange fellow and enjoy seeing practical aspects of research help make a positive change. |
| It is an area of great interest to me, as I have wide-ranging PPI experience in the area of AI. |
| I have been working as a public adviser for 7 years first for CLAHRC NWC and now ARC NWC. I have a genuine interest in AI and would like to explore about it as I come from a south Asian background and can bring voice from my community. I am interested in exploring the challenges of translating Artificial intelligence into practice in health and care settings. |

"
